# Visualizing heavy fermion confinement and Pauli-limited superconductivity in layered CeCoIn_5_

**DOI:** 10.1038/s41467-018-02841-9

**Published:** 2018-02-07

**Authors:** András Gyenis, Benjamin E. Feldman, Mallika T. Randeria, Gabriel A. Peterson, Eric D. Bauer, Pegor Aynajian, Ali Yazdani

**Affiliations:** 1 Joseph Henry Laboratories of Physics, Department of Physics, Princeton University, Princeton, NJ 08544 USA; 2 Los Alamos National Laboratory, Los Alamos, NM 87545 USA; 3 Department of Physics, Applied Physics and Astronomy, Binghamton University, Binghamton, NY 13902 USA; 4 Present Address: Department of Electrical Engineering, Princeton University, Princeton, NJ 08544 USA; 5 Present Address: Department of Physics, Stanford University, Stanford, CA 94305 USA; 6 Present Address: National Institute of Standards and Technology, Boulder, CO 80305 USA

## Abstract

Layered material structures play a key role in enhancing electron–electron interactions to create correlated metallic phases that can transform into unconventional superconducting states. The quasi-two-dimensional electronic properties of such compounds are often inferred indirectly through examination of bulk properties. Here we use scanning tunneling microscopy to directly probe in cross-section the quasi-two-dimensional electronic states of the heavy fermion superconductor CeCoIn_5_. Our measurements reveal the strong confined nature of quasiparticles, anisotropy of tunneling characteristics, and layer-by-layer modulated behavior of the precursor pseudogap gap phase. In the interlayer coupled superconducting state, the orientation of line defects relative to the *d*-wave order parameter determines whether in-gap states form due to scattering. Spectroscopic imaging of the anisotropic magnetic vortex cores directly characterizes the short interlayer superconducting coherence length and shows an electronic phase separation near the upper critical in-plane magnetic field, consistent with a Pauli-limited first-order phase transition into a pseudogap phase.

## Abstract (web summary)

The electronic properties along the out-of-plane direction of layered materials are often inferred indirectly. Here, Gyenis et al. directly probe in cross-section the quasi-two-dimensional correlated electronic states of the heavy fermion superconductor CeCoIn_5_.

## Introduction

A central theme of the research on unconventional superconductivity has been its strong relationship to reduced dimensionality^1–4^. For example, the layered crystal structure of high-*T*_c_ superconductors gives rise to strongly two-dimensional (2D) electronic behavior, which increases the many-body correlation effects that are an essential ingredient for unconventional superconductivity. The heavy fermion superconductor CeCoIn_5_, which has many similarities to the high-*T*_c_ cuprates^5–10^, also has a layered crystal structure built up from the heavy fermion antiferromagnet CeIn_3_^11^ separated by CoIn_2_ stacks. Bulk measurements of CeCoIn_5_ show signatures of an anisotropic, quasi-2D electronic structure^12–17^, but in contrast to the cuprates, there are also contributions from 3D bands that result in a smaller electronic anisotropy^18^. Among the Ce-based heavy fermion compounds, CeCoIn_5_ has the highest transition temperature at ambient pressure, which correlates with its electronic dimensionality as illustrated by isovalent substitutions^19–21^ and layer engineering^22,23^. Like the cuprates, superconductivity in CeCoIn_5_ has a ${\boldsymbol{d}}_{{\boldsymbol{x}}^2 - {\boldsymbol{y}}^2}$ symmetry^24–31^ and there are indications of a pseudogap phase^24,30,32–35^ as well as other ordered phases that compete or coexist with superconductivity, such as the spin-density wave order identified as the *Q*-phase^36–38^. This phase appears at high magnetic fields, just before the upper critical field associated with a Pauli-limited transition into the pseudogap phase^30,39,40^.

Here we introduce an experimental approach to investigate the electronic structure of CeCoIn_5_: we use a scanning tunneling microscope (STM) to study its properties in cross-section. Our measurements directly probe the layer dependence of the electronic states, and represent the first cross-sectional study of a layered superconducting system. Our approach reveals important features of the correlated quasi-2D electronic structure in CeCoIn_5_, including confinement of heavy quasiparticles on the atomic scale and the layer dependence of its pseudogap. Furthermore, in the superconducting state, the cross-sectional geometry enables us to probe the direction-dependent response of the ${\boldsymbol{d}}_{{\boldsymbol{x}}^2 - {\boldsymbol{y}}^2}$ order parameter to scattering from defects as well as spatially resolve the nature of the vortex phase and its first-order Pauli-limited phase transition into the pseudogap state.

## Results

### Layered crystal structure of CeCoIn_5_

**Fig. 1:**
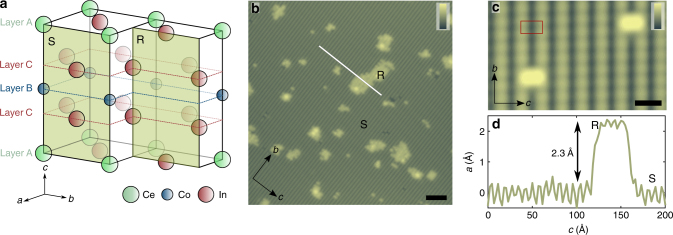
STM topographic images of the [100] surface of CeCoIn_5_. **a** Schematic diagram of the bulk crystal structure of CeCoIn_5_ showing the two possible surface terminations (S and R) when cleaving along the [100] orientation. Lines indicate the positions of layers A, B, and C. The lattice constants are *a* = *b* = 4.6 Å and *c* = 7.52 Å. **b** Constant current topographic image (*V*_bias_ = −100 mV, *I*_setpoint_ = 1.2 nA) of the [100] surface, which displays a large atomically ordered surface S and small islands of the reconstructed surface R. Scale bar: 50 Å; color scale: 7 Å. **c** Topographic image of a few unit cell area on surface S with red rectangular showing a unit cell on the *b–c* plane. Scale bar: 10 Å; color scale: 2 Å. **d** Topographic linecut along the white line indicated on **b**, which shows the height difference between surface S and R and corresponds to *a*/2 = 2.3 Å

To probe the quasi-2D nature of electronic behavior in the normal and superconducting phases of CeCoIn_5_, we cleave samples along the [100] orientation (parallel to the *b–c* surface) in situ in an ultra-high vacuum STM. Based on the crystal structure, we expect that the resulting surfaces expose a cross-sectional cut of the quasi-2D layers of this compound (Fig. 1a). The crystal structure also suggests that the surface termination in the [100] orientation will be either a Ce-Co-In_2_ layer or an In_3_ layer, and STM topographical images indeed show two different surfaces for the cleaved samples in the *b*–*c* plane (Fig. 1b). One is atomically ordered and smooth, and we label it surface S; the other appears more disordered, and we refer to it as surface R. We identify surface R as the In_3_ layer and attribute the quasi-ordered bumps in the STM images to surface reconstruction (see the details in Supplementary Note 1 and Supplementary Fig. 1). We assign the atomically ordered surface S to the Ce-Co-In_2_ layer (Fig. 1c), which is expected to be offset from surface R with a step height of half of the lattice constant in the *a–b* plane, as found experimentally (Fig. 1d). The morphology of the surfaces allows us to identify the position of the quasi-2D layers (in previous studies^30,41,42^ referred as layer A and B) based on topographic images (Supplementary Note 2 and Supplementary Fig. 2). Our measurements of surface S reveal an anisotropic atomic lattice consistent with the crystal structure of CeCoIn_5_, and we focus on high-resolution measurements of this surface in the remainder of this work.

### Signatures of quasiparticle confinement in the normal state

**Fig. 2:**
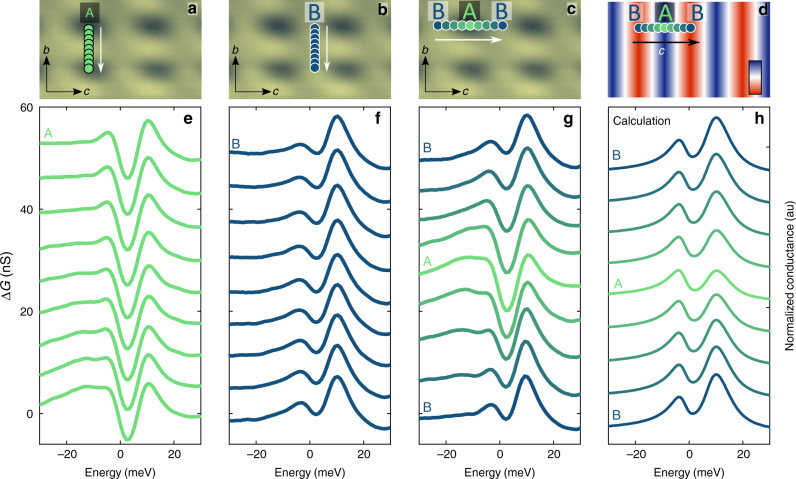
Atomic scale variation of the fermion mass. **a**–**c** Topographic images with color-coded dots indicating the spatial positions where the STM tunneling spectra were acquired (with corresponding colors in **e**–**g**; *V*_bias_ = −100 meV and *I*_setpoint_ = 1.7 nA). In **a**,** b** the spectra were obtained along lines parallel to the *b-*axis that are one lattice constant long (*b* = 4.6 Å). They display negligible spatial variation (**e**, **f**) and correspond to light (layer A) and heavy mass (layer B), respectively. **c**, **g** Tunneling spectra across a one lattice constant long (*c* = 7.5 Å) line parallel to the *c*-axis between two consecutive B layers showing alternating peak–dip structure and indicating that the observed electron mass varies with the position in the unit cell. The same smooth background is subtracted from all spectra and the curves are vertically shifted for clarity. **d** The spatial dependence of the modeled tunneling sensitivity ratio (*t*_*f*_*/t*_*c*_), where *t*_*f*_ and *t*_*c*_ describes the sensitivity of tunneling into the heavy and light part of the excitations, respectively. Color scale shows the ratio from −0.54 to −0.3. **h** Calculated tunneling spectra along the *c-*axis

Our ability to access CeCoIn_5_ layers in cross-section provides a unique opportunity to address the role of the layered structure of this compound on its electronic properties. Previous STM studies^30,41,42^ of CeCoIn_5_ on samples cleaved along the [001] orientation had been used to examine the layer dependence of the electronic properties by studying multiple surfaces perpendicular to the *c*-axis that were terminated with different layers. In those experiments, it was found that the STM tip couples differently to the heavy or light quasiparticles depending on the surface atomic termination, resulting in changes in the tunneling spectra. When tunneling is sensitive to the light *spd* electrons (on layer A), the spectra show a hybridization gap for such quasiparticles, whereas on layer B a stronger coupling to the *f*-electrons yields an asymmetric double peak in the spectra associated with the formation of the dispersing heavy *f*-band. In the current work, spectroscopic measurements on the exposed *b–c* plane enable us to investigate the composite nature of electronic states and their variation in the different quasi-2D layers while studying a single atomic surface (Fig. 1a). Measurements on surface S at *T* = 10 K show two types of spectra depending on the atomic positioning of the tip (Fig. 2): one corresponding to layer A (Fig. 2a, e), in which a hybridization gap is observed and the other to layer B (Fig. 2b, f), where a double peak feature is resolved. One type of spectrum evolves smoothly into the other as the STM tip examines the layers of CeCoIn_5_ in cross-section (Fig. 2c, g). This smooth progression illustrates the remarkable property that the observed electron mass varies significantly on the atomic scale within a single [100] unit cell and that it is strictly associated with the 2D layers. The transition between light and heavy nature of the excitations can be captured by a simple model (Fig. 2d, h) that considers the spatial dependence of the tunneling sensitivity (see Methods section).

One intriguing observation is that the spectroscopic signatures of the *spd* electron hybridization depends on whether the tunneling occurs perpendicular or parallel to the 2D layers. Previous measurements performed on the *a–b* plane^30,31,41^ indicate the presence of two gap-like features in the tunneling spectra (with energy scales of around 40 meV and 15 meV). These can be associated with a direction-dependent hybridization gap (or gaps^43,44^) based on quasiparticle interference (QPI) measurements^41^. In contrast, in our current cross-sectional experiments, we only observe one feature that matches the smaller hybridization gap when tunneling perpendicular to the same layer (Fig. 2e). Our data show that in addition to the previously observed in-plane anisotropy of the measured hybridization gap, the geometry of the STM measurement strongly influences the sensitivity of such measurements.

**Fig. 3:**
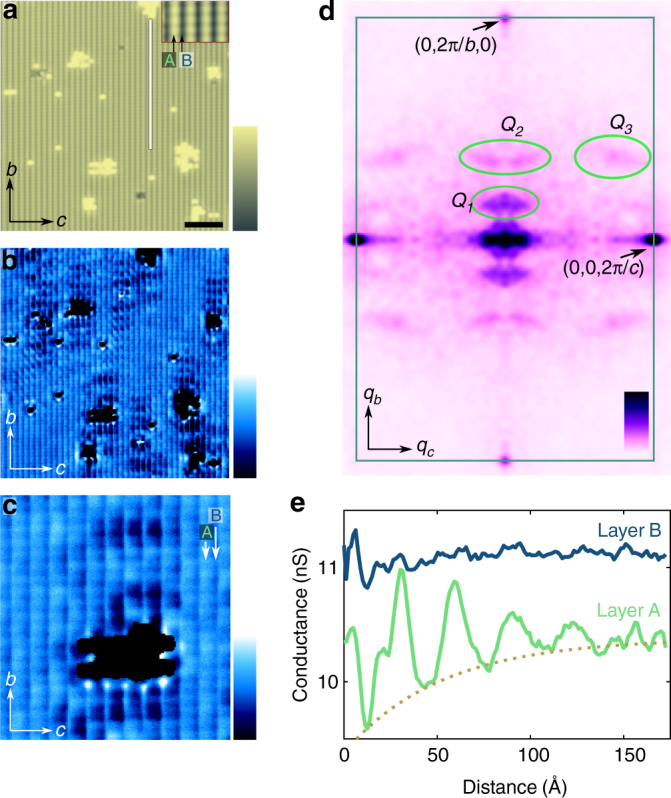
Quasiparticle interference on the [100] surface. **a** Topographic image of surface S where the conductance map was acquired (*V*_bias_ = −70 mV, *I*_setpoint_ = 1 nA). Inset shows an enlarged topographic image with the position of layer A and B indicated. Scale bar: 50 Å; color scale: 5 Å. **b** Conductance map at *E* = −70 meV energy showing quasiparticle standing waves around the atomic islands. The conductance of the islands is artificially saturated for clarity. Color scale indicates the conductance and ranges from 9 to 12 nS. **c** Enlarged conductance map, which demonstrates the strongly one-dimensional scattering of the quasiparticles. Arrows indicate the position of layers A and B. Scale bar: 20 Å; color scale: conductance from 5 to 7 nS. **d** Symmetrized Fourier transform of the conductance maps shown in **b**. Green rectangle shows the border of the unit cell in reciprocal space. Color scale indicates the magnitude of the power spectral density. **e** The modulation of the LDOS along a line parallel to *b*-axis (shown as white line in **a**) on top of layer B (blue) and top of layer A (green). Dark yellow curve shows the exponential decay envelope of the interference pattern obtained by fitting the data with $G\left( d \right) = G_0\sin \left( {\frac{{2\pi }}{{\lambda _{{\mathrm{QPI}}}}}d + \varphi } \right)e^{ - d/\xi _{{\mathrm{QPI}}}} + G_{{\mathrm{mean}}}$, where *d* is the distance from the island, $\lambda _{{\mathrm{QPI}}} = 31$ Å is the wavelength of the quasiparticle signal, $\xi _{{\mathrm{QPI}}} = 52.4$ Å is the decay length, $\varphi$ is the phase of the signal, and $G_{{\mathrm{mean}}}$ is the mean conductance

Mapping variations of the local density of states (LDOS) in the tunneling spectra on the *b–c* surface provides evidence for strong confinement of quasiparticles within the quasi-2D layers of CeCoIn_5_. Figure 3a shows a region where several islands of surface R act as scattering potentials, giving rise to modulations in the LDOS from QPI^45^ (Fig. 3b). Far from the defects (for example, at the bottom left corner of Fig. 3a, b) the QPI signal is absent and the LDOS exhibits a periodic modulation along the *c*-axis. This is the same behavior as observed in Fig. 2, and it further demonstrates that the stacked quasi-2D layers have different electronic characters. Near the islands, our cross-sectional imaging geometry reveals a preferential direction for quasiparticle scattering: the interference waves are oriented along the *b*-axis, whereas the modulation is almost absent in the direction of the *c-*axis. This strongly confined scattering behavior can be further demonstrated by taking a Fourier transform of the conductance map (Fig. 3d), which reveals three significant scattering vectors. The *Q*_1_ vector with the strongest intensity and the weaker *Q*_2_ are in the [010] direction and correspond to scattering along the quasi-2D layers (along the *b*-axis). The presence of 3D bands in CeCoIn_5_ leads to a scattering vector *Q*_3_, which has both [010] and [001] components (with $Q_3 \approx \left( {0,0.37,0.69} \right)$ relative lattice units), although this scattering signal is substantially weaker than the in-plane signal at *Q*_1_. We note that no scattering vector can be detected purely in the [001] direction, which indicates the low probability of electrons moving perpendicular to the quasi-2D layers (in the direction of the *c-*axis).

High-resolution conductance mapping (Fig. 3c) illustrates an additional aspect of the confinement of the quasiparticles: the strength of the QPI signal is strongly suppressed on lines on top of layer B. This is also visible in Fig. 3e, which displays the QPI modulation as a function of distance from the island on two neighboring atomic planes (one is on top of layer A and the other one is on top of layer B). On layer A, the interference signal exhibits a decaying, periodic, long-wavelength modulation of about 30 Å, whereas it is almost absent on layer B, showing that the dominant scattering vector is mainly detectable on layer A. Energy-resolved QPI measurements along the [010] direction (Supplementary Note 3 and Supplementary Fig. 3) reveal two major bands (a heavy and a light band) and shows that the long-wavelength signal (*Q*_1_) at this energy arises from scattering involving light bands, and hence its prominence on layer A is consistent with our discussion above and previous STM measurements ^30,31,41,42^.

### Unconventional superconductivity in cross-section

**Fig. 4:**
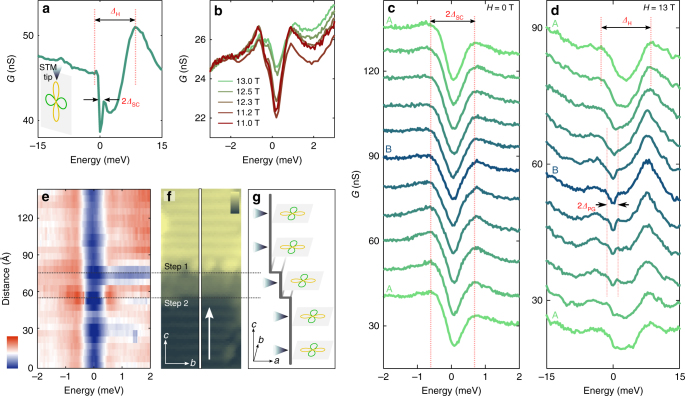
Superconductivity and pseudogap phase in [100] CeCoIn_5_. **a** Averaged tunneling spectra (*V*_bias_ = −30 meV, *I*_setpoint_ = 1 nA) obtained in the superconducting phase at *T* = 400 mK, exhibiting a sharp superconducting gap (${{\Delta }}_{{\mathrm{SC}}}$) around the Fermi energy. Inset: schematic picture showing the relative position of the STM tip and the superconducting order parameter. **b** Averaged tunneling spectra (*V*_bias_ =  −20 meV, *I*_setpoint_ = 500 pA) acquired in high magnetic field around H* show an abrupt jump of the zero-bias conductance between 12.3 T and 12.5 T. **c** High energy-resolved measurement (*V*_bias_ = −6 meV, *I*_setpoint_ = 300 pA) of the superconducting gap along a one unit cell long line parallel to the *c-*axis with the color of the curves indicating the position of the spectra on the S surface (green corresponding to the top of layer A and blue to the top of layer B). **d** Tunneling conductance measurement (*V*_bias_ = −30 meV, *I*_setpoint_ = 1 nA) along a line on S surface above the superconducting transition at *H* = 13 T, which reveals a layer-dependent pseudogap (${{\Delta }}_{{\mathrm{PG}}}$) opening around layer B, whereas the spectra on layer A exhibits only the hybridization gap (${{\Delta }}_{\mathrm{H}}$). **e** The superconducting gap evolution (*V*_bias_ = −10 mV, *I*_setpoint_ = 500 pA) along a 140 Å long line through a double atomic step edge as indicated on the topographic image in **f**. The superconducting gap is insensitive to the potential variation due to the step edge. Color scale in **e** shows the conductance from 25 to 40 nS, while in **f** it refers to the height of the topographic image from 0 to 6 Å. **g** Schematic picture of the position of the *d*-wave order parameter and the STM tip along the step edge

Next, we study the superconducting state of CeCoIn_5_, where our cross-sectional geometry allows us to map the antinodal direction of the ${\boldsymbol{d}}_{{\boldsymbol{x}}^2 - {\boldsymbol{y}}^2}$ order parameter, which points out of plane from the exposed surface (Fig. 4a, inset). When the sample is cooled to $T \approx 400$ mK, well below its transition temperature, the spectrum exhibits a sharp, superconducting gap at the Fermi energy (Fig. 4a), which is unchanged as the STM tip crosses the quasi-2D layers (Fig. 4c), reflecting the fact that the coherence length is much longer than the interlayer spacing. The gap size $\Delta _{{\mathrm{SC}}} \approx$ 550 µeV is similar to previously measured values^25,28,30,31^. Tunneling into a layered *d*-wave superconductor in cross-section has not been previously demonstrated; our measurements offer a new approach for studying its response to impurities. Examining the spatial variation of the gap in the *b–c* surface, we find no variation in $\Delta _{{\mathrm{SC}}}$ across atomic step edges (Fig. 4e–g), in stark contrast to a previous measurement of scattering events in the *a–b* plane^30^. In that experiment, suppression of the superconducting gap was observed due to the sign change of the order parameter for electrons and holes with different in-plane momenta. In our geometry, we find that the gap is insensitive to such defects, which is consistent with the *b–c* surface of CeCoIn_5_ having a *d*-wave order parameter with a uniform phase (see schematic in Fig. 4g).

Application of a magnetic field induces vortices and eventually quenches superconductivity through a first-order phase transition to create a pseudogap state in CeCoIn_5_^24,30,32–35^. We first discuss our STM spectroscopic measurements which reveal signatures of superconductivity up to a magnetic field H*, which is higher than the upper critical field *H*_c2_ obtained from bulk thermodynamic studies^36^. The evolution of the spectra with magnetic field (measured between vortices, see below) is shown in Fig. 4b. There is a jump in the zero-bias conductance between 12.3 T and 12.5 T, which is associated with a first-order transition, in this case out of the superconducting state into a pseudogap state. Similar jumps in the spectra were reported in a previous study for the field applied along the *c*-axis^30^. However, this H* = 12.3 T transition field is above the bulk *H*_c2_ = 11.8 T measured with thermodynamic techniques in CeCoIn_5_ samples from the same batch. Differences between measurements of *H*_c2_ from transport and thermodynamic studies have been previously reported in related heavy fermion systems (see Methods section and refs.^46–48^). While we currently do not have a full explanation for this apparent difference between the STM-measured H* and the bulk *H*_c2_ values, our STM data suggest that superconductivity survives locally to fields larger than the bulk *H*_c2_.

Unlike the superconducting state, the pseudogap phase of CeCoIn_5_ shows a layer-dependent behavior similar to the confined electronic nature of the normal state discussed above. The LDOS exhibits pronounced variations on the atomic scale, as shown in Fig. 4d for *H* = 13 T (which is above the bulk *H*_c2_ and surface measured H*). In layer A, the spectrum resembles the normal state at zero field and displays only the hybridization gap; in contrast, layer B exhibits an additional suppression of conductance over a smaller energy range around the Fermi level, indicative of a pseudogap. These results are consistent with previous observations that the pseudogap in CeCoIn_5_ is observed only when tunneling into the layer B, where there is strong coupling to *f*-electrons^30^. By imaging in cross-section, we not only confirm that the pseudogap feature is associated with the layers exhibiting heavy electronic behavior but also demonstrate that this phase varies on the atomic scale on a single cleaved surface, in sharp contrast to the superconducting phase. Observation of a spectroscopic signature of a pseudogap is consistent with transport studies of CeCoIn_5_^24,32,34,35^, although there has been effort to explain this observation based on a heavy quasiparticle band structure effect^49^.

### Vortex lattice transition of a Pauli-limited superconductor

**Fig. 5:**
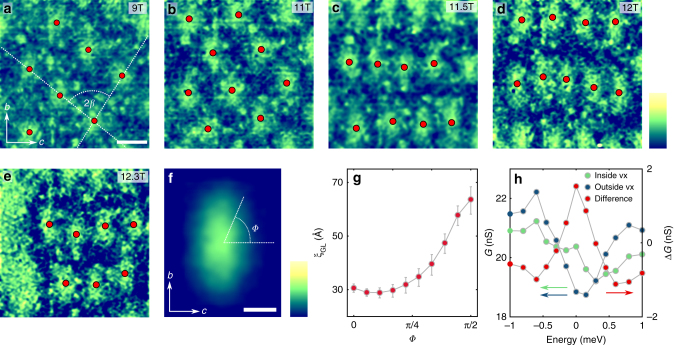
Anisotropic vortices and vortex lattice transition. **a**–**e** Subtracted conductance maps (*G*_sub_) obtained on a 500 Å x 500 Å area with magnetic fields applied parallel to the *a*-axis, which show elongated vortices on the [100] surface. Red dots indicate the fitted centers of mass of the vortices. Dashed line displays the fit through the centers of mass of the vortices to determine the opening angle *β*. The color scale corresponds to the normalized subtracted conductance map $G_{{\mathrm{sub}},{\mathrm{norm}}} = G_{{\mathrm{sub}}}/$ $\left| {\overline {G_{{\mathrm{sub}}}} } \right|$, where $\overline {G_{{\mathrm{sub}}}}$ is the mean of the subtracted conductance value over the entire field of view. It ranges from −2 to 2. Scale bar: 100 Å. **f** Averaged vortex shape obtained by overlaying 90 measured vortices at different fields. $\phi$ corresponds to the angle with respect to the *c-*axis. Scale bar: 30 Å; color scale indicates $G_{{\mathrm{sub}},{\mathrm{norm}}}$ from −1 to 1. **g** Extracted effective coherence length as a function of angle $\phi$, with error bars estimated from the uncertainty of the $G\left( {r,\phi } \right)\sim e^{ - r/\xi \left( \phi \right)}$ fit. **h** Spatially averaged density of states (see Supplementary Fig. 6 for details) in the vortex core (green), far from the vortex (blue), and their difference (red), which show the existence of the bound states inside the vortex

In the presence of a magnetic field, the superconducting state develops vortices, and our cross-sectional technique allows us to visualize the anisotropy of the electronic behavior in the resulting vortex state of CeCoIn_5_. By probing vortices in the *b–c* plane, we extract a direction-dependent characteristic coherence length, map the unusual vortex lattice structure, and directly image the transition of a Pauli-limited superconductor. A series of maps obtained in the same area between 9 T and 12.3 T are shown on Fig. 5a–e (also see Supplementary Fig. 5), where the lighter elongated regions of high conductance correspond to vortex cores, and the red dots represent the fitted center of mass of each vortex. We present background subtracted conductance maps to suppress the effect of conductance variations due to different surface terminations and defects in the field of view (Supplementary Note 4, Supplementary Fig. 4 and ref.^50^). Although the shape of individual vortices is disordered due to surface inhomogeneity and impurities, they exhibit an overall ellipsoid shape. To suppress the effects of inhomogeneity, we overlay all (~90) vortices measured at various fields through their center of mass. The resulting average vortex displays an azimuthally asymmetric core (Fig. 5f), which is a manifestation of the anisotropic coherence length in the *b–c* plane. Although a detailed model calculation of the local density of states that includes the multiband nature of CeCoIn_5_ is needed to fully characterize the vortex core shape, we extract characteristic lengths from our data by fitting the decay of the vortex conductance as function of distance *r* from the center at different angles $\phi$ with respect to the *c*-axis according to $G( {r,\phi } )\!\sim\! e^{ - r/\xi ( \phi )}$^51^. From this fit, we find characteristic lengths of $\xi ^c =$ 30 Å along the *c*-axis to $\xi ^b =$ 65 Å along the *b-*axis (Fig. 5g), which are consistent with values estimated for the in- and out-of-plane coherence lengths from measurements of the angle dependence of *H*_*c2*_^14^. Conductance maps taken at various energies confirm the presence of the zero bias peak inside the vortex core (Fig. 5h and Supplementary Fig. 6).

Our high magnetic field measurements in the *b–c* plane demonstrate an unusual structural transition in the vortex lattice which is different from the ones found when the magnetic field was applied in the [001] or [110] direction^52–54^. As illustrated in Fig. 5, for *H* < 11 T, the vortices are arranged in a distorted hexagonal Abrikosov lattice with a field-independent *β* = 41 ± 2° opening angle, in excellent agreement with small angle neutron scattering studies^54^. However, when the magnetic field is increased above 11 T, a previously unreported vortex lattice transition occurs. In this phase, the vortices are arranged in rows along the *c*-direction, with larger spacing along the *a*-axis. One possible cause of such a change of the vortex lattice could be the onset of the Q-phase. However, such transition in the vortex lattice could also result from various effects such as the strong local anisotropy of the vortices, nonlocal electrodynamic effects between them, or superconducting gap symmetry effects^55^.

Finally, by mapping the electronic structure in close proximity of H* we directly image the transition of a Pauli-limited superconductor to its normal state^39,40,53^. Generally, two effects of the applied magnetic field govern the physics of a superconducting condensate: the kinetic energy of the supercurrent around the vortices and the Pauli energy of the electron spins coupled to the external field. In an orbital limited superconductor, the superconductivity is suppressed by the overlap of vortices, while in a Pauli-limited case, the Cooper pairs are destroyed by breaking the spin-singlet state, as is the case in CeCoIn_5_. Imaging the vortex state near the critical field at H* = 12.3 T (Fig. 5e) shows the coexistence of a normal region and vortices in the same field of view, while above H* only normal regions are present (Supplementary Fig. 5). Due to the short anisotropic coherence length, the distance between the cores and the orbiting supercurrents is large, which allows the Pauli-paramagnetic effects to dominate the orbital effects in CeCoIn_5_. Moreover, the emergence of domains is expected to occur for first-order phase transitions; the coexistence of both normal and superconducting regions therefore provides a direct visualization of the first-order superconducting phase transition in CeCoIn_5_.

## Discussion

In conclusion, we have explored the influence of the layered material structure and reduced effective dimensionality of CeCoIn_5_ on its confined electronic properties by utilizing the STM as a cross-sectional probe for samples cleaved along the [100] direction. Spectroscopic measurements performed in the normal and superconducting states demonstrate the effects of quasi-two-dimensionality, from varying effective electron mass on the atomic scale and confined quasiparticle scattering to layer-dependent pseudogap behavior and anisotropic vortex structure in the superconducting state. Imaging these dramatic effects in cross-section offers a direct illustration of quasi-2D electronic behavior in this archetypal correlated electron system.

## Methods

### Sample growth and preparation

The single-crystal samples used for the measurements were grown from excess indium at Los Alamos National Laboratory. Crystals with large thickness in the *c-*direction were chosen for the measurements, cut into suitable sizes (with dimensions in all directions of ~0.5–2 mm), oriented, and glued to the sample holder with the [100] surface facing up. An aluminum post with the same horizontal dimension was glued to the top of the sample and used to cleave the sample along the *c*-axis in ultra-high vacuum at room temperature. Immediately after cleaving the samples, they were inserted into our home-built STMs. We used a variable temperature STM for the *T* = 10–20 K temperature measurements and a dilution fridge STM for the low temperature ($T \approx 400$ mK) and high magnetic field experiments. A large number of samples (around 30) were cleaved in both setups, and each cleaved sample was approached multiple times (using long range piezoelectric motion). On the cleaved samples, we found atomically flat surfaces suitable for STM measurements with a success rate around 10% of the approaches. Differential conductance measurements were performed using standard lock-in techniques, with voltage bias applied to the sample.

### Theoretical model of the tunneling density of states

To capture the spectroscopic features (Fig. 2), we use a theoretical model^56,57^, which was previously successfully applied to data acquired on the *a–b* surface of CeCoIn_5_^30,41^. In this model, the differential conductance d*I*/d*V* can be obtained from the interference of tunneling paths into two channels: the light and heavy electronic excitations.

The dispersion of the conduction band is

$${\it{\epsilon }}_{\boldsymbol{k}} = 2t(\cos k_x + \cos k_y) - \mu$$

whereas for the heavy band it is

$$\chi _k = - 2\chi _0(\cos k_x + \cos k_y) - 4\chi _1\cos k_x\cos k_y + {\it{\epsilon }}_f$$

where *k*_*x*_ and *k*_*y*_ are the wavevectors, *t* is the nearest neighbor hopping, $\mu$ is the chemical potential, $\chi _0$ and $\chi _1$ correspond to the antiferromagnetic correlation between the *f* moments, and ${\it{\epsilon }}_f$ can be associated with the chemical potential for the *f*-electrons.

The components of the full Green’s function are

$$G_{ff}({\mathbf{k}},\omega ) = \left\{ {\left[ {G_{ff}^0\left( {{\mathbf{k}},\omega } \right)} \right]^{ - 1} - s^2G_{cc}^0\left( {{\mathbf{k}},\omega } \right)} \right\}$$

$$G_{cc}\left( {{\mathbf{k}},\omega } \right) = \left\{ {\left[ {G_{cc}^0\left( {{\mathbf{k}},\omega } \right)} \right]^{ - 1} - s^2G_{ff}^0\left( {{\mathbf{k}},\omega } \right)} \right\}^{ - 1}$$

$$G_{cf}\left( {{\mathbf{k}},\omega } \right)= - G_{cc}^0\left( {{\mathbf{k}},\omega } \right)sG_{ff}\left( {{\mathbf{k}},\omega } \right),$$

where *s* describes the coupling between the magnetic moments and the conduction electrons and $G_{ff}^0\left( {{\mathbf{k}},\omega } \right) = \left( {\omega - \chi _{\boldsymbol{k}} + i\Gamma _f} \right)^{ - 1}$, $G_{cc}^0\left( {{\mathbf{k}},\omega } \right) = \left( {\omega - \varepsilon _{\boldsymbol{k}} + i\Gamma _c} \right)^{ - 1}$ with the corresponding inverse lifetimes of $\Gamma _f$ and $\Gamma _c$.

The *dI/dV* spectrum can be approximated as

$$\frac{{{\mathrm{d}}{I}\left( {{\mathbf{r}},\omega } \right)}}{{{\mathrm{d}}{V}}} \propto - {\mathrm{Im}}\mathop {\sum }\limits_{i,j = 1}^2 \left[ {\hat t\hat G\left( {{\mathbf{r}},\omega } \right)\hat t} \right]_{ij}$$

where $$\hat t = \left[ {\begin{array}{*{20}{c}} {t_c} & 0 \\ 0 & {t_f} \end{array}} \right]$$ describes the sensitivity to tunnel into heavy or light part of the electrons.

In our calculation, we use $t = 200$ meV, $\mu = 2t$, $\chi _0 = 0.01t$, $\chi _1 = 0.06\chi _0$, ${\it{\epsilon }}_f = 0.035t$, $s = 0.15t$, and $\Gamma _c = \Gamma _f = 0.015t$, and vary the $t_f/t_c$ ratio as a function of position with respect to the two-dimensional layers (Fig. 2d, h).

### The upper critical field identified by different techniques

Our STM measurements carried out in magnetic fields applied in the [100] direction show the absence of the signatures of superconductivity at the field of H* = 12.3 T, which is higher than the previously reported upper critical field *H*_c2_ values^36^. Our samples are of high quality and show bulk thermodynamic *H*_c2_ = 11.8 T, consistent with many other previous studies. Here, we discuss possible reasons for the experimental observation that superconductivity locally survives above the bulk *H*_c2_.

We first emphasize that our STM measurements clearly show that there is a superconducting gap (measured outside of the vortices), which evolves smoothly from lower fields, and survives up to 12.3 T (Fig. 4b). The difference between the superconducting gap and the pseudogap is clear in our measurements, as there is a jump in the zero energy conductance between 12.3 T and 12.5 T. Spectroscopic imaging as a function of field (Fig. 5) also clearly shows the vortex lattice surviving through the bulk *H*_c2_, and the lack of overlap between the vortices is consistent with the Pauli-limited nature of superconductivity in this compound. Furthermore, the observation of coexisting normal regions and superconducting areas with vortices at 12.3 T is consistent with a first-order superconducting phase transition.

A second important point is that in the CeMIn_5_ (M = Co, Rh, Ir) superconductors, one often finds a significant difference between the upper critical field determined from bulk measurements (e.g., specific heat, nuclear magnetic resonance) compared to *H*_c2_ determined from transport measurements (see, for example refs.^46–48^). Usually, this difference between *H*_c2_^transport^ and *H*_c2_^bulk^ occurs when antiferromagnetism is present above the superconducting transition. CeIrIn_5_ is a notable exception, with no obvious antiferromagnetic transition observed above the bulk superconducting transition at *T*_c_^bulk^ = 0.4 K, while *T*_c_^transport^ = 1.3 K with a corresponding difference in *H*_c2_^bulk^ = 0.9 T < *H*_c2_^transport^ = 7 T for fields applied in the *a*–*b* plane^48^. Based on the similarities of CeCoIn_5_ to CeIrIn_5_ (i.e., no antiferromagnetism present above *T*_c_ in zero magnetic field) and on our experimental findings (presence of the vortex lattice and evidence of the superconducting gap from d*I*/d*V* measurements) we conclude that superconductivity in CeCoIn_5_ is observed up to H* = 12.3 T (*H*||*a*).

Our STM measurements, which are uniquely sensitive to the electronic structure on the surface, are the first local measurements to provide insight into the superconducting properties of CeCoIn_5_ near *H*_c2_ for fields applied in the [100] direction. We hope that this result will stimulate further work to understand the origin of the discrepancy in measured upper critical field from different techniques.

### Data availability

The data that support the findings of this study are available from the corresponding author upon reasonable request.

## Electronic supplementary material

Supplementary Information
