# Supplementary material for: Visualizing heavy fermion confinement and Pauli-limited superconductivity in layered CeCoIn5
Source: Nat Commun. 2018 Feb 7;9:549. doi: 10.1038/s41467-018-02841-9 (PMC5803268; doi:10.1038/s41467-018-02841-9)
Supplement: Supplementary file 1 — Supplementary Information [file 41467_2018_2841_MOESM1_ESM.pdf]

Supplementary Figure 1

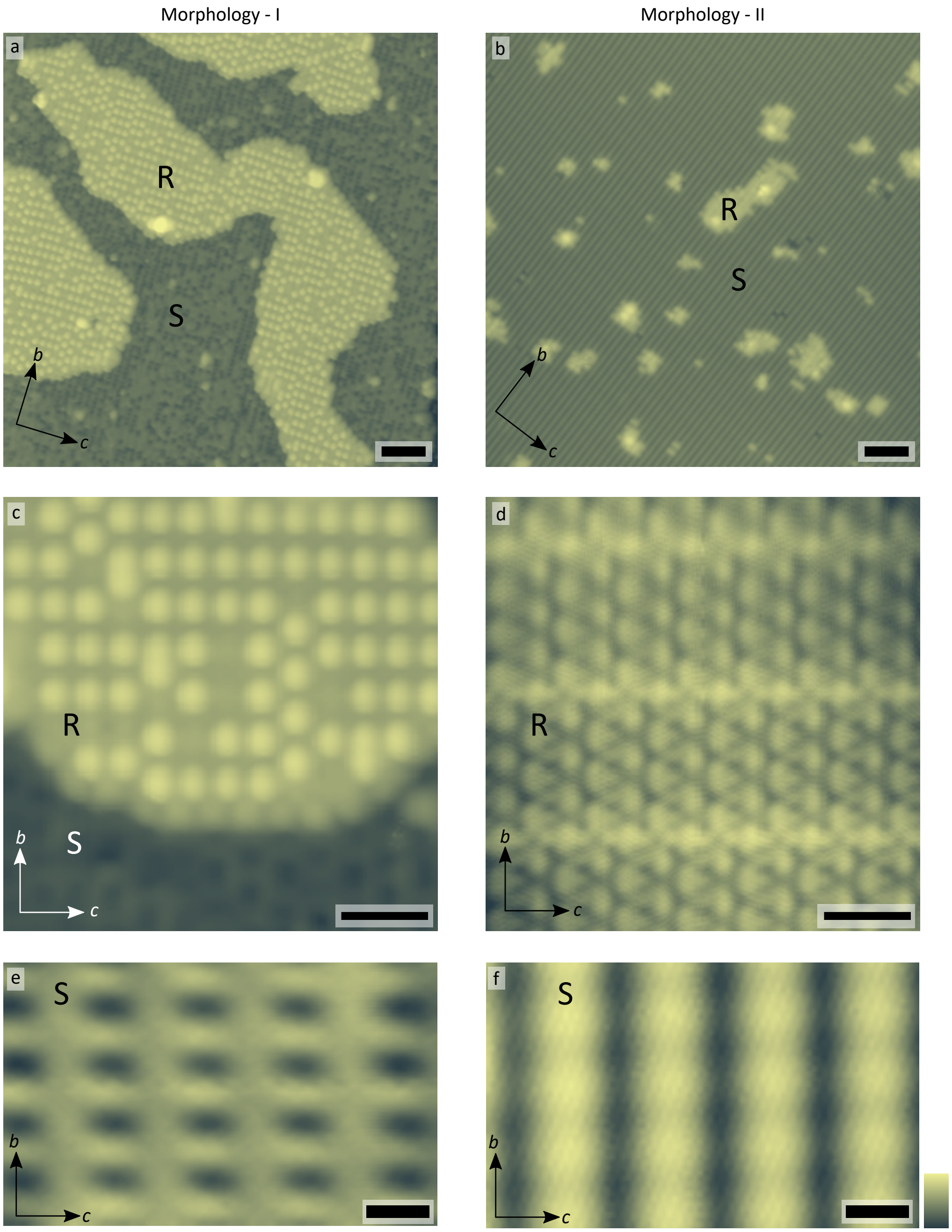

Supplementary Figure 1. **STM topographic images of the observed surface morphologies of (100) $CeCoIn_5$.** a-b, Constant current topographic images (V_{bias} = - 60 mV, $I_{setpoint}$ = 100 pA and V_{bias} = - 100 mV, $I_{setpoint}$ = 1.2 nA) of the two observed types of surface morphologies, which display the consecutive reconstructed surface R and atomically ordered surface S. c-f, Enlarged topographic images of surface R and surface S in the case of the two morphologies. Scale bars correspond to the following lengths (in order from panel a to f): 50 Å, 50 Å, 20 Å, 20 Å, 5 Å, 5 Å, while vertical color scales correspond to the following heights: 7 Å, 7 Å, 4 Å, 1 Å, 0.2 Å, 1.5 Å.

Supplementary Figure 2

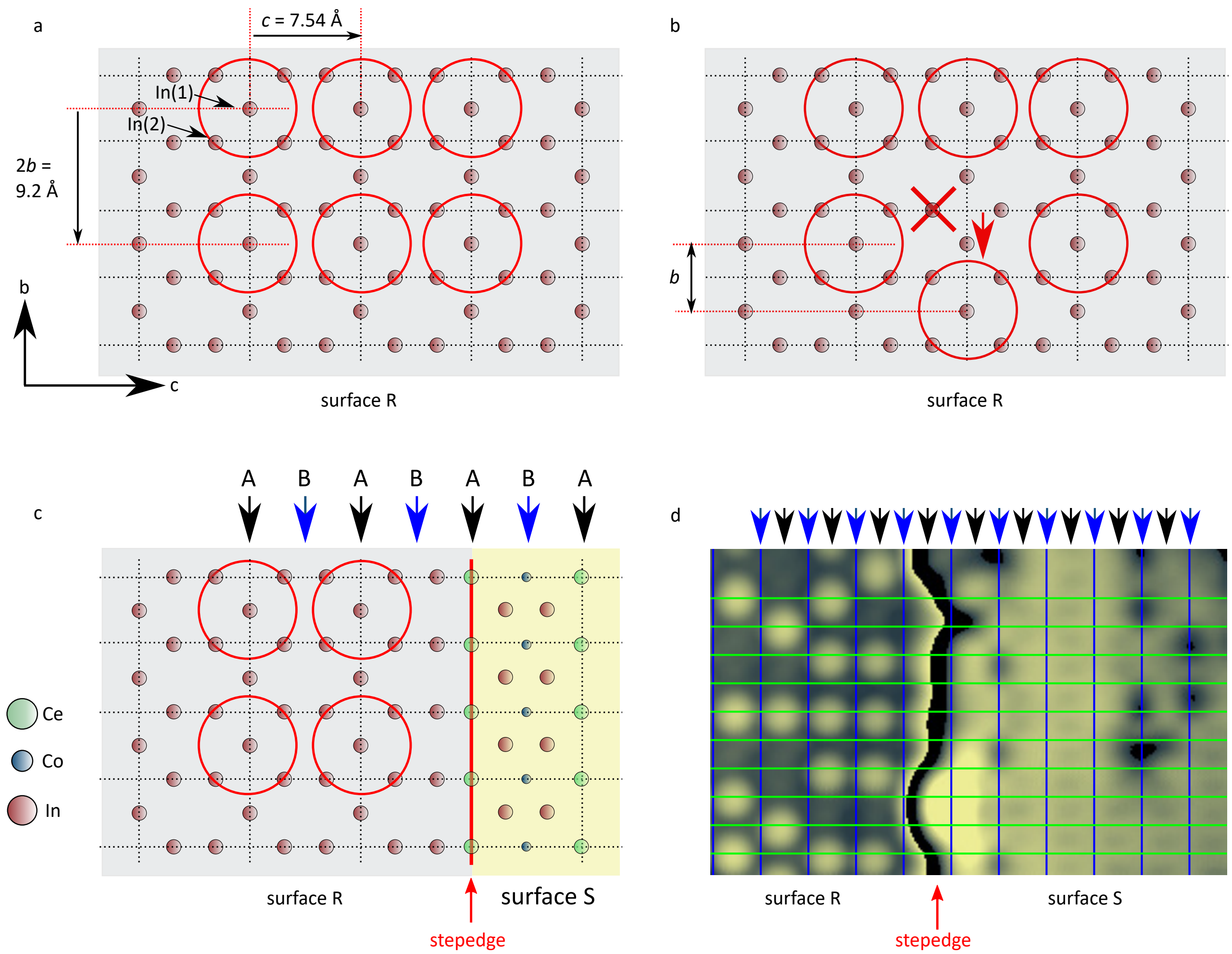

Supplementary Figure 2. **Identification of the position of the quasi-two-dimensional layers.**
a, The In-terminated layer R on the *b*-*c* surface of $CeCoIn_5$. Due to surface reconstruction, five In atoms (highlighted with red circles) form the circular objects observed in the STM images. The quasi-lattice of the reconstruction has 2*b* x *c* quasi-periodicity. b, When one In atom is absent (e.g., due to the cleaving procedure), the corresponding reconstructed sphere is shifted by a lattice constant in the *b* direction. c, The center of the circular superstructures corresponds to the position of layer A (black arrows), and their edges correspond to layer B (blue arrows). d, Topographic image of R and S surfaces separated by a single step edge, showing the identified layers (blue lines and arrows correspond to layer B, black arrows to layer A). The horizontal green lines indicate the lattice in the *b* direction.

Supplementary Figure 3

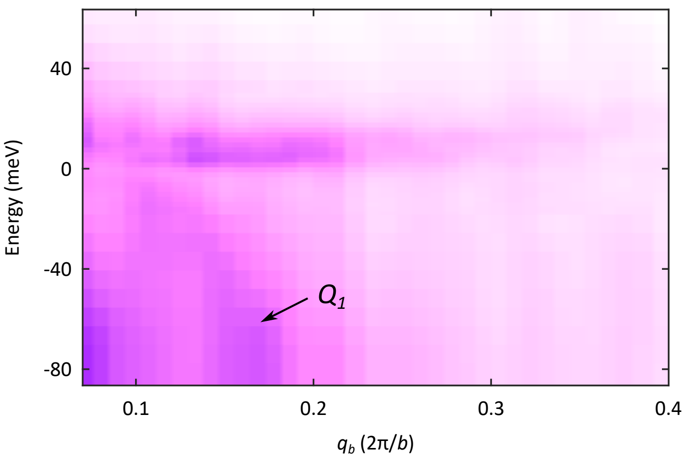

Supplementary Figure 3. **Energy-momentum structure of the quasi-particle interference.** Fourier transform amplitude (in the [010] direction) of the conductance maps obtained at different energies shows two pronounced features. At larger negative energies (from -10 meV to -80 meV), the Q_1 vector (around 0.16 r.l.u.) slowly disperses, which indicates that it originates from the light conduction band. Around the Fermi energy, a rapidly dispersing signal appears, which is the result of scattering between the heavy bands.

Supplementary Figure 4

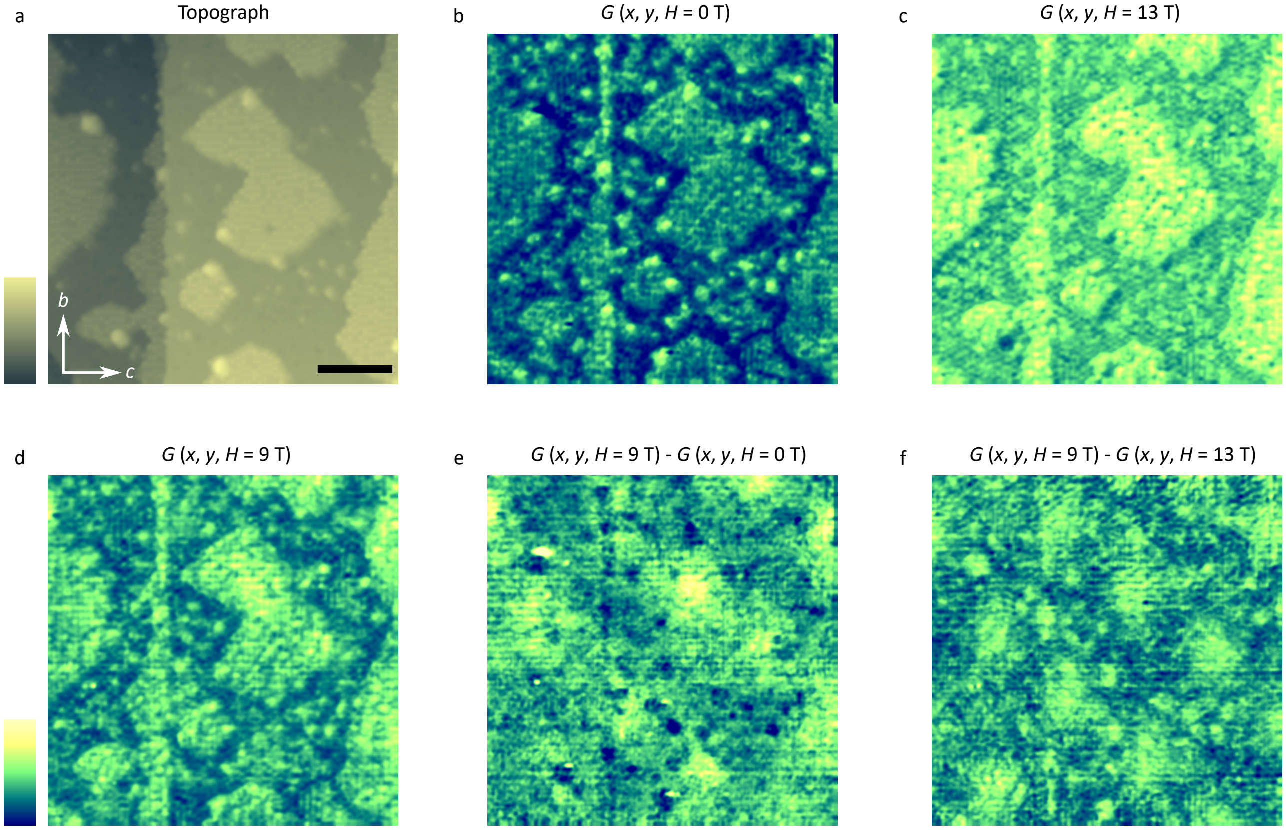

***Supplementary Figure 4.* Comparison of subtracted conductance maps in high magnetic fields.**
a, Topographic image of the 500 Å x 500 Å large area on the *b-c* surface of $CeCoIn_5$, where all the vortex maps presented in this work were obtained. Scale bar: 100 Å; color scale: 15 Å. b-d, Conductance maps at various magnetic fields. Color scale indicates the conductance and ranges from 15 to 45 nS.
e-f, Subtracted conductance maps using H = 0 T and H = 13 T as reference fields; color scales indicate subtracted conductance from -2 to 14 nS, and from - 9 to 7 nS, respectively.

Supplementary Figure 5

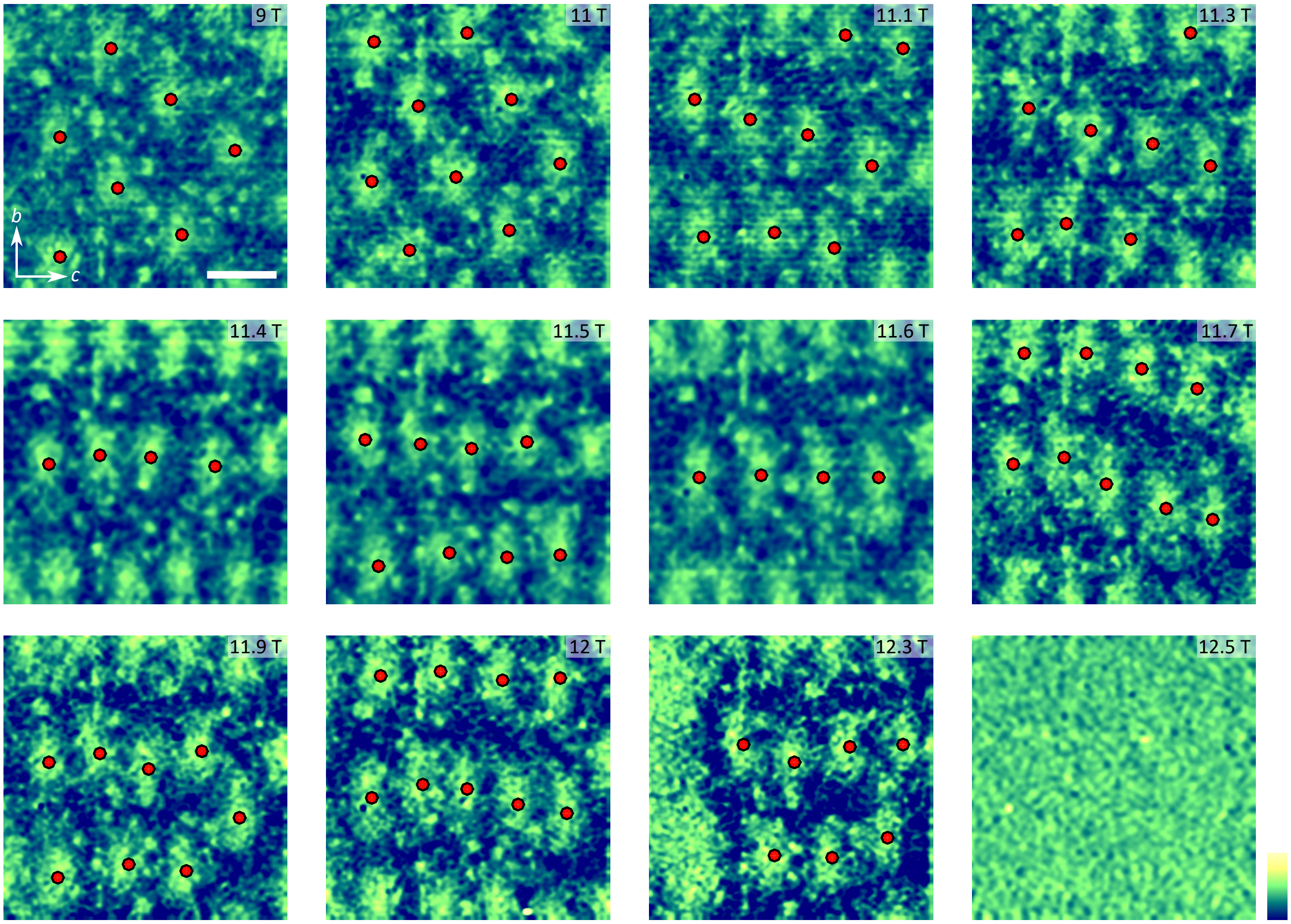

Supplementary Figure 5. **Vortices at high magnetic fields on the *b*-*c* surface of $CeCoIn_5$.**
Subtracted conductance maps (G_{sub}) at various fields in the vicinity of the upper critical field (V_{bias} = - 10 mV, $I_{setpoint}$ = 300 pA; reference field: H = 13 T). Scale bar corresponds to 100 Å; the color scale shows $G_{norm,sub}$ from -2 to 2. Red dots indicate the fitted center of mass of the vortices.

Supplementary Figure 6

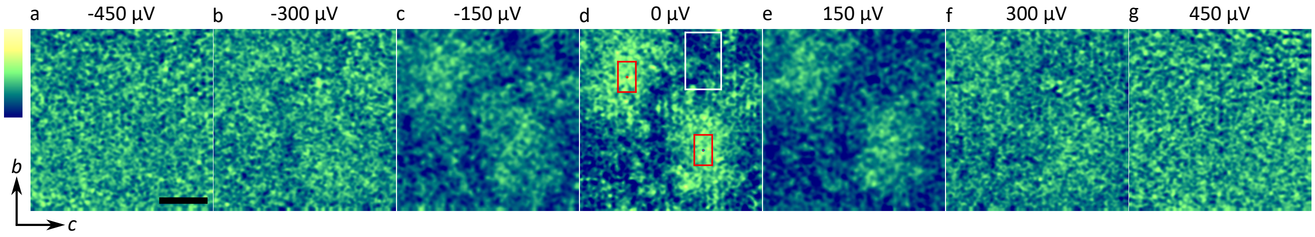

***Supplementary Figure 6.* Energy structure of the vortices and bound states.**
a-g, Subtracted conductance maps at different energies showing that the vortices are most visible at zero energy. The red and white rectangles show the spatial positions where the averaged conductance values were obtained at each energy for inside and outside the vortex core, respectively (Fig. 5h). Scale bar: 50 Å; color scale: G_{sub} from -2 to 4 nS.

Supplementary Note 1. Observed morphologies of the (100) cleaved surface

We cleaved several samples along the [100] direction, approached multiple different areas on each cleaved sample, and consistently found two distinct surface morphologies (Supplementary Fig. 1). For most regions we approached, the topographic images revealed morphology-I (Supplementary Fig. 1a), whereas in rare cases morphology-II (Supplementary Fig. 1b) was observed. In both cases, we found two surface terminations, one of which (surface R) is reconstructed (Supplementary Fig. 1c-d). We associate this surface with the layer containing In atoms (In_3 layer) because previous measurements on $CeCoIn_5$ cleaved along the (001) direction (*1*) also showed a reconstructed In_2 surface. Surface S in the second kind of morphology shows a rectangular lattice with lines running in the high symmetry directions (Supplementary Fig. 1f), whereas in the first kind of morphology it shows a mixture of rectangular (Supplementary Fig. 1e) and hexagonal order (Supplementary Fig. 1c). The topography in the hexagonal regions is inverted relative to the reconstructed surface but has identical lattice spacing. We therefore believe that it either reflects the influence of a subsurface In_3 layer or results from the detailed atomic arrangement after the cleave. On surface S, not all atoms in the unit cell are visible, but the structural modulation resembles the (100) unit cell, and the position of the two-dimensional layers can be indirectly determined (see Supplementary Note 2). Although the origin of the two different morphologies is unclear, the normal vector of the surfaces, the in-plane atomic modulations, and the step heights are in good agreement with the expected crystal structure of the (100) plane of $CeCoIn_5$. We also note that both types of morphologies have been observed on a single cleaved sample.

Supplementary Note 2. Origin of the reconstructed In surface and determination of the position of the 2D layers

The Ce, Co, and In atoms are not all visible on the (100) cleaved surfaces, so we use an indirect method to determine the position of the 2D atomic layers based on the unusual structure of the reconstructed In_3 surface. As Supplementary Fig. 2a shows, two inequivalent In atoms exist on surface R: In(1) (from layer A) and In(2) (from layer C). By examining the quasi-ordered reconstructed surfaces observed in our STM topographic images, we conclude that the simplest model to explain the pattern involves a superstructure containing one In(1) atom and four surrounding In(2) atoms. This assignment matches well to both the size and the orientation of the features on the reconstructed surface of the more common morphology. Supplementary Fig. 2b shows that the superstructure lattice can shift by half of its unit cell in one direction,

corresponding to a shift by one unit cell in the *b* direction; this may occur spontaneously or in response to a missing In atom on the surface. As a result, we observe superstructures running in the *b* direction with frequent instances of shifts within these lines.

Since the In(1) atom is part of layer A, we determine that layer A is located along the midpoint of the large circular superstructures, whereas layer B is located between them (Supplementary Fig. 2c). By following these 2D planes through a step edge, we can also identify the layers on surface S (Supplementary Fig. 2c-d). The assignment of the 2D layers based on the reconstructed surface is consistent with the assignment based on spectroscopic features that we observe on each layer (double peak or gap in the spectrum in Figs. 2e-g).

Supplementary Note 3. Energy-momentum dispersion and lifetime of the quasiparticle interference signal

In Supplementary Fig. 3, we show the amplitude of the Fourier transform of differential conductance as a function of energy and momentum in the [010] direction. The data illustrate that Q_1 is slowly dispersing ($v \approx 0.8\ \mathrm{eV\AA}$) at energies far from the Fermi level and can be associated with the light part of the hybridized band structure. We also observe a flat band around zero energy corresponding to the enhanced scattering of heavy excitations similar to measurements on $CeCoIn_5$ cleaved along the [001] direction (*1-4*). The measured decaying QPI signal (Fig. 3e) and the dispersion relation allow us to extract the lifetime of the quasiparticles. As discussed in the main text, fitting the amplitude of the modulation to an exponential decay function yields a decay length $\xi_{\mathrm{QPI}} = 52\ \mathrm{\AA}$. Based on the measured dispersion relation, this results in a lifetime of $\tau_{\mathrm{QPI}} = \xi_{\mathrm{QPI}}/v \approx 40$ fs. This value is in good agreement with the quasiparticle lifetime obtained from the $\Delta E = 10$ meV width of the spectral function measured on surface B (*1*), from which $\tau = \hbar/\Delta E \approx 65$ fs.

Supplementary Note 4. Conductance maps in magnetic field

As we discuss in the main text, we use a background subtraction scheme to enhance the visibility of the vortices. We define the subtracted conductance maps as $G_{\mathrm{sub}}(x, y, E, H) = G(x, y, E, H) - G(x, y, E, H_{\mathrm{ref}})$, where $G(x, y, E, H)$ is the real space conductance value acquired at energy E, magnetic field H at spatial position of (x, y), while H_{ref} corresponds to the reference magnetic field (*5*). As a reference, we choose

conductance maps obtained at H_{ref} = 13 T ($H_{ref} > H^*$) instead of zero field because the magnetic field dramatically reduces some of the impurity scattering resonances (*6*). We note that using H = 0 T as reference leads to qualitatively similar results. Finally, we choose E = 0 because the sharpest vortex imaging contrast is achieved at the Fermi energy (Fig. 5h and Supplementary Fig. 6). We use a drift correction code to compensate for the small displacements of the acquired conductance maps at different magnetic fields.

In Supplementary Fig. 4, we plot the raw conductance maps (Supplementary Fig. 4b-d) and the subtracted maps (Supplementary Fig. 4e-f) obtained on the surface shown in Supplementary Fig. 4a. The maps show that the average conductance varies significantly on the different R and S surfaces. Nonetheless, the subtraction allows us to image the vortices. Choosing the H = 0 T map as a reference leads to dark, circular regions associated with impurity scattering resonances (Supplementary Fig. 4e), which are absent in the subtracted map where H = 13 T is the reference field (Supplementary Fig. 4f).

In addition to the data presented in the main text, we show subtracted conductance maps at other magnetic fields (Supplementary Fig. 5) and at other energies (Supplementary Fig. 6).

Supplementary References

[1] Aynajian, P. *et al.* Visualizing heavy fermions emerging in a quantum critical Kondo lattice. *Nature* **486,** 201–206 (2012).
[2] Zhou, B. B. *et al.* Visualizing nodal heavy fermion superconductivity in $CeCoIn_5$. *Nat. Phys.* **9,** 474–479 (2013).
[3] Allan, M. P. *et al.* Imaging Cooper pairing of heavy fermions in $CeCoIn_5$. *Nat. Phys.* **9,** 468–473 (2013).
[4] Gyenis, A. *et al.* Quasi-particle interference of heavy fermions in resonant x-ray scattering. *Sci. Adv.* 2 (10), e1601086 (2016).
[5] Hoffman, J. E. *et al.* A four unit cell periodic pattern of quasi-particle states surrounding vortex cores in $Bi_2Sr_2CaCu_2O_{8+\delta}$. *Science* **295,** 466–469 (2002).
[6] Yazdani, A., Howald, C. M., Lutz, C. P., Kapitulnik, A., & Eigler, D. M. Impurity-induced bound excitations on the surface of $Bi_2Sr_2CaCu_2O_8$. *Phys. Rev. Lett.* **83,** 176–179 (1999).
